# Fibroblasts Express Immune Relevant Genes and Are Important Sentinel Cells during Tissue Damage in Rainbow Trout (*Oncorhynchus mykiss*)

**DOI:** 10.1371/journal.pone.0009304

**Published:** 2010-02-18

**Authors:** Hans-Christian Ingerslev, Carlo Gunnar Ossum, Thomas Lindenstrøm, Michael Engelbrecht Nielsen

**Affiliations:** 1 Section for Aquatic Protein Biochemistry, Division for Seafood Research, DTU Food, National Food Institute, Lyngby, Denmark; 2 Department of Biochemistry, August Krogh Institute, University of Copenhagen, Copenhagen, Denmark; 3 Department of Infectious Disease Immunology, Statens Serum Institut, Copenhagen, Denmark; Centre de Recherche Public de la Santé (CRP-Santé), Luxembourg

## Abstract

Fibroblasts have shown to be an immune competent cell type in mammals. However, little is known about the immunological functions of this cell-type in lower vertebrates. A rainbow trout hypodermal fibroblast cell-line (RTHDF) was shown to be responsive to PAMPs and DAMPs after stimulation with LPS from *E. coli*, supernatant and debris from sonicated RTHDF cells. LPS was overall the strongest inducer of IL-1β, IL-8, IL-10, TLR-3 and TLR-9. IL-1β and IL-8 were already highly up regulated after 1 hour of LPS stimulation. Supernatant stimuli significantly increased the expression of IL-1β, TLR-3 and TLR-9, whereas the debris stimuli only increased expression of IL-1β. Consequently, an *in vivo* experiment was further set up. By mechanically damaging the muscle tissue of rainbow trout, it was shown that fibroblasts in the muscle tissue of rainbow trout contribute to electing a highly local inflammatory response following tissue injury. The damaged muscle tissue showed a strong increase in the expression of the immune genes IL-1β, IL-8 and TGF-β already 4 hours post injury at the site of injury while the expression in non-damaged muscle tissue was not influenced. A weaker, but significant response was also seen for TLR-9 and TLR-22. Rainbow trout fibroblasts were found to be highly immune competent with a significant ability to express cytokines and immune receptors. Thus fish fibroblasts are believed to contribute significantly to local inflammatory reactions in concert with the traditional immune cells.

## Introduction

Throughout the distant evolutionary lineage from the very primitive invertebrates to the phylogenetically more ‘modern’ vertebrates, an inflammatory reaction is established when tissue is damaged or infection is initiated [1]–[4]. The types of cells and the signalling molecules taking part in this process are dependent on the evolutionary position of the organism [5]. Among the vertebrates, the inflammatory response is in general characterised by recruitment of cells like polymorphnuclear neutrophilic leucocytes, monocytes and macrophages to the tissue of injury and/or infection [6], [7]. Invasion of these cell types mediate enhanced phagocytosis and expression of pro-inflammatory cytokines as IL-1β, TNF-α, and of numerous chemokines like IL-8 and other CXC chemokines [8]-[10]. The inflammatory immune reaction may not only be elicited by ‘traditional’ immune cells like macrophages and neutrophils that are recruited or already present in the tissue. Several reports suggests that in humans, the conventional non-immune cell-type fibroblast, also have immune regulating capabilities [11]–[13] and can thus serve as highly important sentinel cells [14]. Fibroblasts are the least specialised member of the connective tissue family, whose main function is to maintain the structural integrity of connective tissue by secreting precursors of extracellular matrix rich of type I and/or type-III collagen [15]. At the same time, human fibroblasts are known to express a wide range of cytokines. These include, among others, IL-1β, IL-4, IL-6, IL-8, IL-10, IL-12, TNF-α, IFN-γ, CCL20 and macrophage colony stimulating factor [12], [16]. These cytokines are also known in lower vertebrates like the fish species rainbow trout and zebrafish (*Danio rerio*) [17]-[26]. In addition, human fibroblasts also respond to the secretion of cytokines like IL-1β, TNF-α e.g. by enhancing the production of CCL20 and hyaluronan [12], [27]. Besides expressing various Toll-like receptors (TLRs) [28] these characteristics indicate the presence of several other immune receptors on fibroblasts [12] and subsequent downstream pathways to convey signalling through these [29]–[31]. Further, some human fibroblasts have also been found to be phagocytic and capable of generating respiratory burst reactions as well as being responsive to lipopolysaccharide (LPS) from *E. coli*. [32]–[35]. The sensitivity to LPS most likely relates to the presence of CD14/TLR2/TLR4 receptors on the fibroblast surface, which further indicate that fibroblast serve an important sentinel function through pathogen-associated molecular pattern (PAMP) recognition [11], [32], [36]–[38]. It then seems reasonable that fibroblasts also respond to damage-associated molecular patterns (DAMPs) like necrotic cells and other hydrophobic portions (hyppos) from ‘self’ parts exposed through tissue damage [39].

Whether fibroblasts in lower vertebrates also exhibit phagocytic capacity and/or serve as sentinel cells is currently not known, but previous results from carp (*Cyprinus carpio*) could indicate this. Thirty minutes following injury of the skin, significant up-regulation of CXC receptors, IL-10 and TNF-α was reported [40]. This could indicate the presence of local, responsive cells that were already present in the tissue prior to injury. In lower vertebrates such as fish from the carp family, presence of TLR2 and TLR4 have been shown [41]. However, in evolutionary older fish species like the salmonids, they do not seem to be present although other TLRs have been reported [42]–[44]. Due to the phylogenetic position of fish, they are by choice considered to be an excellent animal to study immune mechanisms since it is the earliest divergent vertebrate, which have both an innate and adaptive immune system [1], [3]. Conversely, the scarce availability of antibodies in teleost research to date often hampers the opportunity for functional experiments. However, the ongoing sequencing of immune genes in fish makes real-time RT-PCR for measuring gene expression a good alternative. As in humans, the adaptive immune system in fish is characterised by the presence of antigen-specific receptors (T cell receptors), immunoglobulin on lymphocytes and MHC class I and II surface molecules [1], [45]. However, the immunoglobulin isotype repertoire in teleost fish is more limited than in e.g. mouse and humans and consists of only IgM, IgD and a unique fish isotype termed IgT [46], [47]. The specific immune cell-repertoire consists of T- and B-cell subpopulations, whereas the non-specific cells consist of phagocytic cells like monocytes/macrophages, granulocytes and natural killer cells [1], [48]. Several different fibroblast cell lines have been characterised from fish. In rainbow trout (*Oncorhynchus mykiss*) at least three fibroblast cell lines termed RTG-2, RTG-P1 and RTHDF exist of where the RTG-2 cell line is expressing the immune genes Mx, iNOS, IL-1β and IL-18 [49]-[55]. In order to examine the immune capabilities of fibroblasts in fish, an *in vitro* assay was setup using the rainbow trout RTHDF cell-line [53]. The cells were subjected to either LPS from *E. coli*, debris or supernatant from sonicated RTHDF cells in order to introduce fibroblast receptors to both PAMP and DAMP stimuli. Further, in order to examine the role of fibroblasts *in vivo*, a model of sterile, mechanical tissue damage of muscle tissue in rainbow trout was established. From both experiments, the muscle tissue and the fibroblasts were subsequently subject to real-time RT-PCR. Expression of the pro-inflammatory cytokines IL-1β and IL-8 as well as the anti-inflammatory cytokine IL-10 was measured since these are key molecules in the initial, inflammatory reaction [56]–[59]. The multifunctional gene transforming growth factor-β (TGF-β) was also included in order to show whether regenerative and proliferative responses were activated following tissue damage. Finally, we examined the expression of the four different TLRs; 3, 5, 9 and 22 in order to show if these were sensitive to PAMPs and/or DAMPs [42], [60], [61]. Together, this study provides new and important information about the role of fibroblasts in lower vertebrates in relation to inflammation, tissue damage and immune competence.

## Materials and Methods

### Culturing of RTHDF Fibroblasts

Rainbow trout hypodermal fibroblasts (RTHDF [53]) were cultured in Leibovitz L-15, supplemented with 15% (w/v) foetal bovine serum (FBS), penicillin (100 units/ml) and streptomycin (100 µg/ml) at 21°C and atmospheric air, as described previously [53]. Trypsin solution for cell detachment was made by dissolving 0.1% (w/v) trypsin and 1 mM disodium EDTA in phosphate-buffered saline, PBS [137 mM NaCl, 2.7 mM KCl, 8.1 mM Na_2_HPO_4_, 1.5 mM KH_2_PO_4_] [62]. All cell culture reagents were used cold from the refrigerator. Cells were subcultured in 25 cm^2^ tissue culture flasks 3 days prior to the experiments and were confluent when harvested. The amount of cells per culture flask when confluent was estimated to 4.5×10^5^ as earlier described [53]. All cell culture reagents were purchased from Life Technologies Inc. (Naperville, IL, U.S.A.) and cell culture plastic wear were purchased from TPR (Trasadingen, Switzerland). Chemicals were from Sigma Aldrich, unless otherwise stated.

### Stimulation of RTHDF Fibroblasts with LPS, Cell-Debris and Supernatant

Confluent 25 cm^2^ cell culture flasks of RTHDF cells containing 3 ml of cell-culture medium were either incubated with sonicated RTHDF fibroblasts, the supernatant from the sonicate or *E.coli* 0111:B4 LPS (Sigma-Aldrich). The sonicate was prepared by an initial trypsination of confluent cell culture flasks followed by sonication for 30 s (amplitude 10 microns) using a MSE Soniprep 150 sonicator (Sanyo). The sonicate was then centrifuged for 2 min at 14.000× g and the clear supernatant was transferred to a new tube. The pellet containing necrotic cells and cell debris was thereafter resuspended in L-15 medium and used for stimulation. The RTHDF fibroblasts were then stimulated in triplicate of cell culture flasks per sampling point. The amount of sonicate added per flask of stimulated cells originated from one flask of RTHDF fibroblasts. The LPS stimulation was performed using 20 µg ml^−1^ of LPS and the amount of supernatant used was 100 µl per cell culture flask. Non-stimulated cells were used as controls. Harvesting of cells for isolation of total RNA was then performed 1 hour, 4 hours and 24 hours post stimulation. This was accomplished by removal of the cell culture medium from the flasks followed by addition of 500 µl of lysis buffer plus 5 µl of β-mercaptoethanol from the GenElute Mammalian™ Total RNA Miniprep Kit (Sigma-Aldrich). The cells were then scraped off the bottom of the flasks with a cell scraper (Greiner Bio-one) and RNA was isolated according to the manufacturers instructions.

### Phagocytic Assay

Phagocytosis of the RTHDF fibroblasts was studied using carboxylate-modified polystyrene fluorescent latex beads with a diameter of 1.0 µm (Sigma-Aldrich) as described by Ganassin *et al*. [63]. A suspension of beads was prepared by adding 2 µl of the commercial latex suspension (2.5% solids latex) to 5 ml of growth medium, which replaced the regular growth medium. The cell-cultures were then observed at 1 hour, 4 hours and 24 hours after incubation and observations were made with a Nikon fluorescence microscope. Prior to observation, the cells were washed four times with PBS in order to wash away adhered, non-ingested beads.

### Rearing Conditions of Mechanically Damaged Fish

Unvaccinated, healthy rainbow trout (*Oncorhynchus mykiss*) reared at Agerskov Dambrug (Bording, Jutland), where delivered to the experimental facilities at the National Institute of Aquatic Resources, Technical University of Denmark at 21 March 2007. The fish were then acclimatised one month prior to mechanical injury and maintained in 200 l plastic tanks with aerated local tap water at 14°C and exposed to a light regime of 16 hours of light followed by 8 hours of dark. Average weight (g ± SD) and length (cm ± SD) at the samplings were 9.5±2.6 and 9.5±0.9, respectively (*n* = 40). At each sampling point five injured fish and five control fish were collected. All procedures were conducted in accordance with the regulations set forward by the Danish Ministry of Justice and animal protection committees by Danish Animal Experiments Inspectorate permit 2007/561-1302 and in compliance with European Community Directive 86/609.

### Procedures for Mechanical Injury and Tissue Sampling

Thirty fish were mechanically injured just below the caudal end of the dorsal fin at April 26 using a home made device containing twenty five sterile needles made from 19G syringes (Becton Dickinson) with a thickness of 1.1 mm equally distributed on an area of 6 mm×6 mm. The needles had a depth of 6 mm to ensure penetration of both the skin and underlying muscle tissue of the fish. Prior to mechanical injury, fish were anaesthetised in MS-222 (50 mg/l) (Sigma-Aldrich). The injury was performed posterior to the dorsal fin above the lateral line and the device was penetrated twice through the skin, giving rise to fifty holes per fish. Prior to sampling of muscle tissue, fish were killed in an overdose of MS-222. Sampling occurred at 4, 8 and 24 hours post injury using a sterile disposable scalpel. The tissue collected was 1) muscle tissue from the site of injury and 2) non-injured muscle tissue from the opposite side of the fish relative to the injury tissue and 3) muscle tissue from non-injured control fish. By sampling the internal control it was possible to show whether the responses in the injured fish were local or systemic. Tissue was collected from five injured and five control fish per sampling point and was transferred to cryo tubes containing RNA *later*® (Sigma-Aldrich) and stored at −20°C until isolation of RNA. For this, fifty mg of the sampled tissues were homogenised by sonication for 30 s (amplitude 10 microns) using a MSE Soniprep 150 sonicator (Sanyo) and RNA was further isolated using a GenElute Mammalian™ Total RNA Miniprep Kit (Sigma).

### CDNA Synthesis

The RNA quality and quantity from the RTHDF cells and the muscle tissue was checked by OD_260/280_ measurements on a GeneQuant II Spectrophotometer (Pharmacia Biotech) and the RNA was finally treated with DNase-I (Sigma-Aldrich) to remove any genomic DNA. Random hexamer primed reverse transcription reactions were performed from 400 ng of total RNA in a 20 µl setup using TaqMan® Reverse Transcription reaction (Applied Biosystems). The synthesised cDNA samples were diluted 1:10 in MilliQ H_2_O and stored at −20°C.

### Quantitative RT-PCR

Quantitative RT-PCR was performed using a Stratagene MX3000P™ real-time PCR system, dual-labelled TaqMan® probes conjugated with either a 5' HEX or a 5' FAM fluorophor, a 3' BHQ_1_ quencher and desalted primers (Sigma-Genosys). The assays for TLR-3, TLR-9 and TLR-22 examined were run using SYBR® Green (Sigma-Aldrich) instead of a probe. The genes chosen for investigation were IL-1β, IL-8, IL-10, TGF-β, TLR-3, TLR-5m (membrane bound form), TLR-9 and TLR-22. For use as an internal control and for normalisation of the results the reference gene ribosomal protein S20 (RPS20) and elongation factor-1α (ELF-1α) were validated for their transcriptional stability in muscle tissue and the RTHDF cells (data not shown). RPS20 was used within muscle tissue since it was more stably expressed between the different individuals and injured versus non-injured fish compared to ELF-1α. For the RTHDF cells, the elongation factor-1α gene was found more suitable [64]. The sequence for the primers and probes, amplicon length and GenBank accession numbers are shown in Table 1. The primers were optimised according to MgCl_2_ and primer concentrations. The cycling conditions for the TaqMan® assay were 94°C for 2 min followed by 40 cycles of 94°C for 15 s and 60°C for 1 min. The cycling conditions for the SYBR® Green assays were the same but the run was terminated by a melting curve analysis where the fluorescence was continually measured during a temperature increase from 60°C to 95°C. Wells for the TaqMan® assays contained 12.5 µl of JumpStart™ *Taq* ReadyMix™ (Sigma–Aldrich), 0.25 µl ROX (Sigma–Aldrich; diluted 10x), 2.5–5.5 mM MgCl_2_ (Sigma–Aldrich), 0.75–1.25 µl forward and reverse primer (10 mM), 1 µl TaqMan® probe (200 nM), 5 µl of diluted cDNA and autoclaved MilliQ water to a volume of 25 µl. Wells for the SYBR® Green assays contained 12.5 µl of SYBR® Green JumpStart™ *Taq* ReadyMix™ (Sigma-Aldrich), 0.25 µl ROX (Sigma–Aldrich; diluted 10x), 3.5–5.5 mM MgCl_2_ (Sigma-Aldrich), 1 µl forward and reverse primer (10 mM), 5 µl of diluted cDNA and autoclaved MilliQ water to a volume of 25 µl. The expression results were analysed using the 2^−ΔΔ*C*t^ method after verification that the primers amplified with an efficiency of approximately 100% (doubling of the product between every cycle in the log-linear phase) and data were shown as −ΔΔ*C*_t_-values and fold expression relative to non-injured control fish or non-stimulated RTHDF cells [65]. The threshold cycle (*C*t) was determined manually and set to 0.01 in the lower level of the log-linear area. The statistical analysis was performed on the Δ*C*_t_ values by a two-tailed T-test in cases of normally distributed data and Mann-Whitney U-test in cases when they were not normally distributed. The statistical software GraphPad Prism version 4.03 was used to calculate the statistics and create graphs.

**Table 1:** Sequences of primers and probes used for the real-time PCR analysis.

Gene	Primer	Sequence (5′-3′)	GenBank acc. no.	Amplicon (bp)
	Forward	ACCCTCCTCTTGGTCGTTTC		
EF-1α	Reverse	TGATGACACCAACAGCAACA	**AF498320**	63
	Probe	GCTGTGCGTGACATGAGGCA		
	Forward	AGCCGCAACGTCAAGTCT		
RPS20	Reverse	GTCTTGGTGGGCATACGG	**NM_001124364**	104
	Probe	TGTGCAGACCTTATCCGTGGAGCT		
	Forward	AGGACAAGGACCTGCTCAACT		
IL-1β	Reverse	CCGACTCCAACTCCAACACTA	**AJ278242**	72
	Probe	TTGCTGGAGAGTGCTGTGGAAGAA		
	Forward	GAGCGGTCAGGAGATTTGTC		
IL-8	Reverse	TTGGCCAGCATCTTCTCAAT	**AJ310565**	72
	Probe	ATGTCAGCGCTCCGTGGGT		
	Forward	GGGTGTCACGCTATGGACAG		
IL-10	Reverse	TGTTTCCGATGGAGTCGATG	**AB118099**	121
	Probe	ATCTCGACACGGTGCTGCCCAC		
	Forward	ACGCCACAGCCAGCTTAG		
TGF-β	Reverse	CGCACACAGCAACTCTCC	**X99303**	87
	Probe	TCTCGGAAGAAACGACAAACCA		
	Forward	ACGGCTCAACCTGAATATGG		
TLR-3	Reverse	GCTCTCCAGTGCCCTTAGTG	**DQ459470**	97
	Probe	----------------------------------------------------		
	Forward	GGCATCAGCCTGTTGAATTT		
TLR-5m	Reverse	ATGAAGAGCGAGAGCCTCAG	**AB091105**	89
	Probe	GCTCAGTCATATCGTGTGAGGAGGA		
	Forward	GCAACCAGTCCTTCCACATT		
TLR-9	Reverse	AAACCCAGGGTAAGGGTTTG	**NM_001129991**	73
	Probe	----------------------------------------------------		
	Forward	AAGGCGCTTCGAGAGTTGAAT		
TLR-22	Reverse	TGGAGAGAGGCTGAAATGATGAG	**AJ628348**	148
	Probe	----------------------------------------------------		

## Results

### Constitutive Expression of Selected Genes

**Figure 1:**
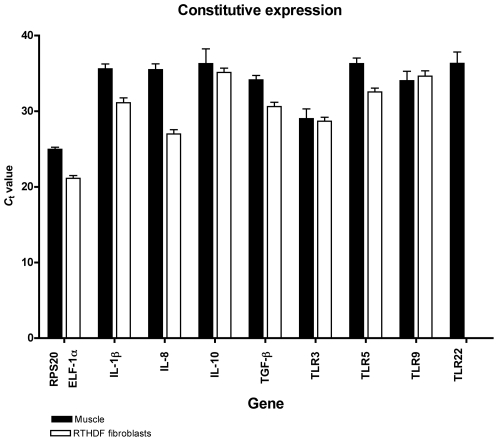
Constitutive expression of the examined genes. The *C*_t_-values for RPS20, ELF-1α, IL-1β, IL-8, IL-10, TGF-β, TLR-3, TLR-5m, TLR-9 and TLR-22 are shown for muscle tissue (black bars) and for RTHDF cells (white bars). The data are presented as mean expression of the control fish from all samples points and mean expression of control RTHDF cells from all sample points. The *C*_t_ value is defined as the threshold cycle number of PCR at which the sample fluorescent signal passes a fixed threshold above the baseline.

All genes except for TLR-22 in the RTHDF cells were expressed within 40 cycles of PCR in non-injured tissue and non-stimulated cells (Figure 1). Except for TLR-9, the constitutive level of expression of the immune genes was higher in the RTHDF cells than in muscle tissue. The highest difference was seen for IL-1β and IL-8 that were approximately 20 and 330 folds higher expressed in the RTHDF cells compared to muscle tissue, respectively. The constitutive expression level of the housekeeping genes was lower for the RPS20 in the muscle tissue compared to the ELF-1α in the RTHDF cells and both genes showed a lower transcriptional variance between individuals/cell replicates than the immune genes.

### Expression in RTHDF Cells following Stimulation with LPS, Debris and Supernatant

**Figure 2:**
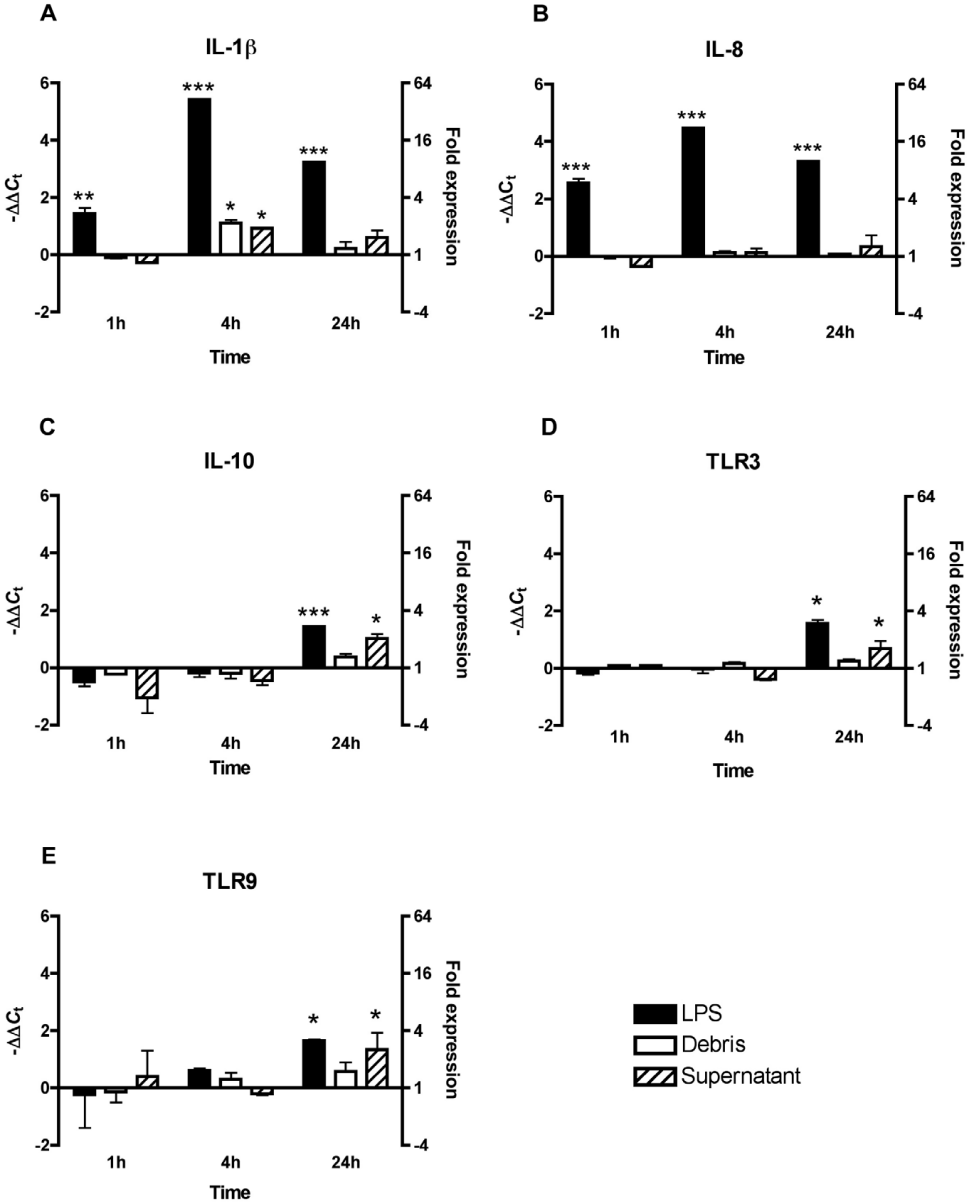
Quantitative real-time PCR for the RTHDF cells. Expression is shown for the genes (A) IL-1β, (B) IL-8, (C) IL-10, (D) TLR-3 and (E) TLR-9. Black bars represent expression in *E.* coli LPS stimulated cells relative to control cells; white bars indicate expression in cells stimulated with debris from sonicated RTHDF cells relative to control cells and striped bars denotes expression in cells stimulated with supernatant from sonicated RTHDF cells relative to control cells. The data are normalised relative to the expression of elongation factor-1α and analysed using the ΔΔ*C*_t_ method. Data are shown as −ΔΔ*C*_t_ values and fold expression. Bars represent mean values of −ΔΔ*C*_t_ + SD values from three cell replicates. * Depicts statistical significance between stimulated cells and control cells (*P<0.05; **P<0.01; ***P<0.001). A −ΔΔ*C*_t_ value of 0 means no regulation relative to control cells.

Figure 2 A-E shows the effect of *E. coli* LPS (0111:B4), debris or supernatant from sonicated RTHDF cells on the expression of IL-1β, IL-8, IL-10, TLR-3 and TLR-9 in the RTHDF cells after 1, 4 and 24 hours of stimulation. No influence on the expression of TGF-β and TLR5m was seen and TLR-22 was not expressed in the RTHDF cells after 40 cycles of PCR (data not shown). Overall the variation in expression between cell replicates was much lower compared to individual fish. The highest effect on all genes was seen for IL-1β and IL-8 after stimulation with LPS. The expression increased significantly after 1 hour of stimulation for both genes peaked after 4 hours to 42.5 and 22 folds, respectively, followed by a decrease in expression to 9.4 and 9.9 folds after 24 hours, respectively (*P*<0.05). The pattern for IL-10 was different and the response was slower than for IL-1β and IL-8. IL-10 was only significantly expressed at 24 hours after stimulation by 2.7 folds relative to non-stimulated cells (*P*<0.05). Stimulation by debris revealed significant impact on only IL-1β, but the response was weak and only significantly elevated at 4 hours post stimulation by 2.2 folds (*P*<0.05). Supernatant from the sonicated RTHDF cells increased the expression of both IL-1β and IL-10, but weakly compared to the effects of LPS. The IL-1β expression increased to 1.9 folds after 4 hours of stimulation, while for IL-10 it was 2 folds after 24 hours relative to non-stimulated cells (*P*<0.05). TLR-3 and TLR-9 showed similar expression patterns. Both LPS and supernatant increased the expression significantly after 24 hours; LPS to approximately 4 fold for both genes and supernatant to 1.6 and 2.5 folds for TLR-3 and TLR-9, respectively (*P*<0.05).

### Phagocytic Assay

The addition of latex beads to the RTHDF cells did not indicate that the cells were able to phagocytose (data not shown). After incubation with beads and washing, a few beads were still attached to the glass slide surrounding the cells, and there was no indication that any beads were taken up and positioned inside the RTHDF cells.

### Mechanical Injury of Muscle Tissue and the Effects on Gene Expression

**Figure 3:**
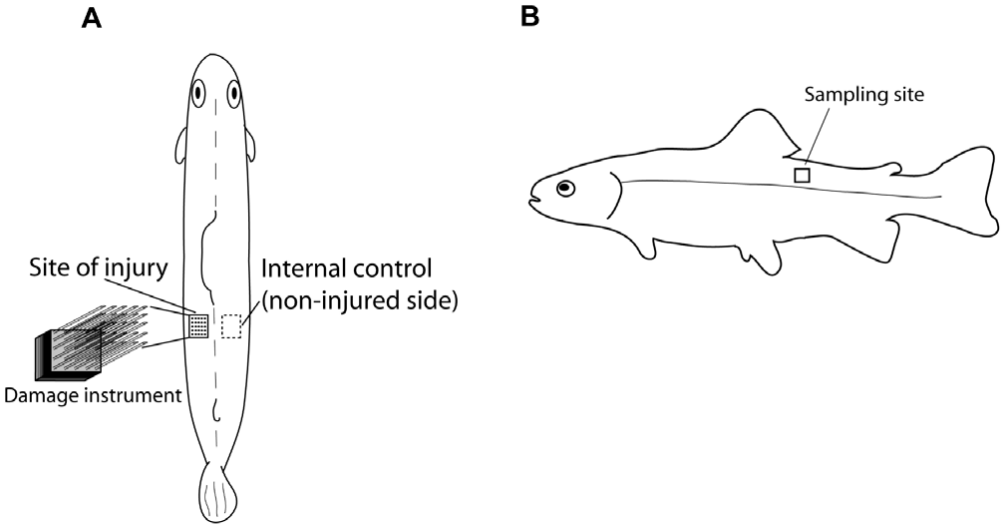
Damage procedures and sampling from mechanically injured rainbow trout. The fish were injured on the left side behind the dorsal fin using the damage instrument. Muscle tissue samples were taken in the injured area while the internal control samples were taken from the same place relative to the dorsal fin on the right side of the fish (A). The vertical position of the site of injury/sampling site is shown in (B). Sampling of muscle tissue from non-injured control fish was performed in the same area as shown for injured fish (not shown).

**Figure 4:**
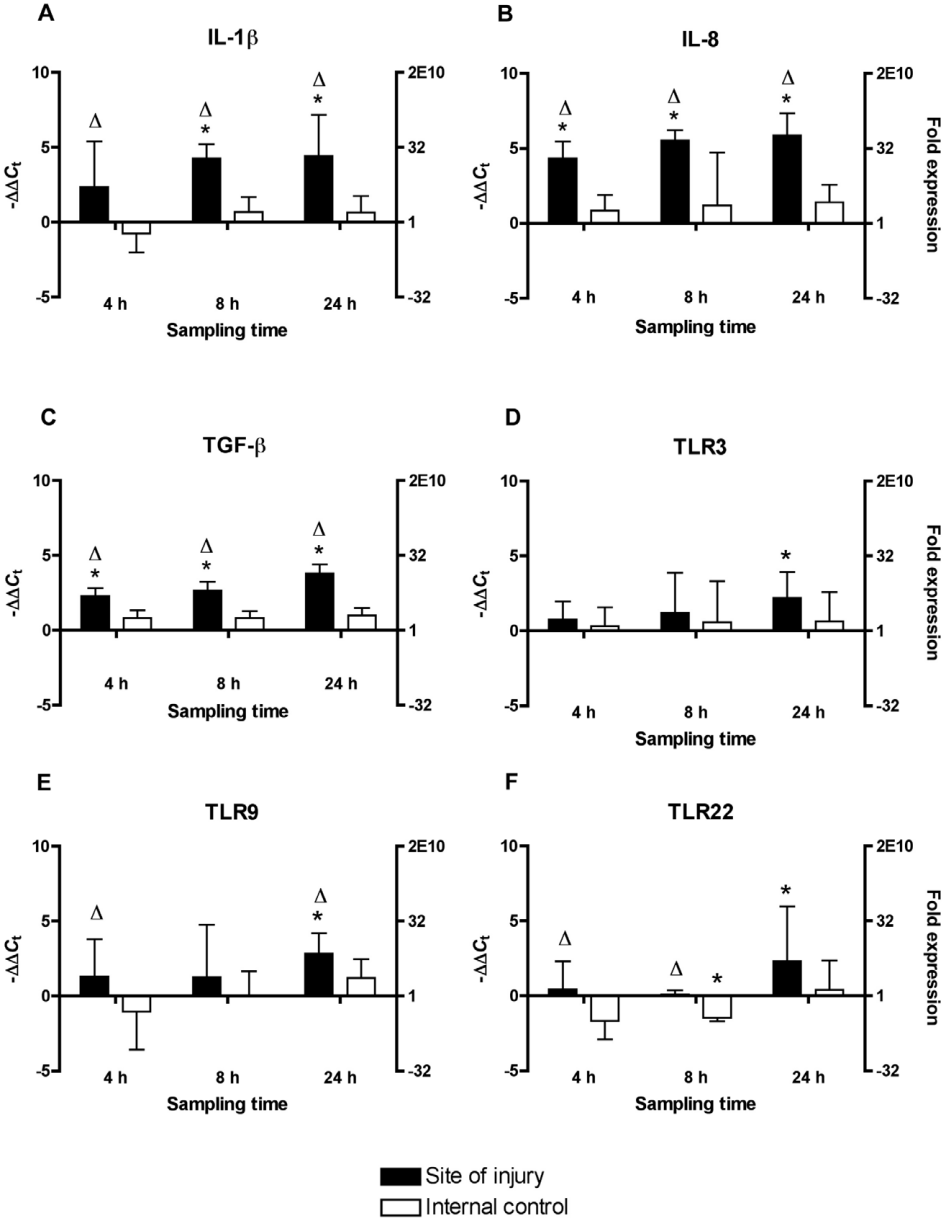
Quantitative real-time PCR for mechanically damaged fish. Expression is shown for the genes (A) IL-1β, (B) IL-8, (C) TGF-β, (D) TLR-3, (E) TLR-9 and (F) TLR-22. Black bars represent expression at the site of injury relative to control fish and white bars indicate expression at the non-damaged internal control site relative to control fish. The data are normalised relative to the expression of ribosomal protein S20 and analysed using the ΔΔ*C*_t_ method. Data are shown as −ΔΔ*C*_t_ values and fold expression. Bars represent mean values of −ΔΔ*C*_t_ + SD values from five individuals. * Depicts statistical significance between injured fish and control fish (*P*<0.05); Δ denotes statistical significant difference between site of injury and internal control site (*P*<0.05). A −ΔΔ*C*_t_ value of 0 means no regulation relative to control fish.

The device used for mechanical tissue damage is shown in figure 3 A–B. The following effects of mechanical injury of the muscle tissue on the expression of the genes IL-1β, IL-8, TGF-β and TLR-3, 5 m, 9 and 22 at the time points 4, 8 and 24 hours post injury (p.i.) is shown in figure 4 A-F. No significant changes were seen for IL-10 (*P*>0.05) (not illustrated). The study showed a strong induction of immune related genes, especially of the cytokines IL-1β, IL-8 and TGF-β in the injured muscle tissue at all samplings. The average up-regulation in the injured fish for the three sampling points was between 5.2 to 21.8 folds for IL-1β; 20.0 to 59.3 folds for IL-8 and 4.9 to 13.9 folds for TGF- β. For all three genes the mean expression level was increasing from 4 hours to 24 hours post injury and the expression at the site of injury was significantly higher at all sampling points compared to the non-injured samples from the same fish (*P*<0.05).

The expression of the TLRs in muscle tissue showed significant changes in the expression for TLR-3, 9 and 22, but not for TLR-5m. TLR-3 was significantly up-regulated at the site of injury 24 hours post damage relative to the control fish corresponding to 4.6 folds while no changes was seen for the internal control samples (*P*<0.05). The expression of TLR-9, however, was significantly up-regulated at the injury site 4 hours and 24 hours by 2.5 and 7.2 folds, respectively (*P*<0.05). TLR-22 showed significant differences between injured and non-injured site 4 hours and 24 hours p.i. indicated by small down-regulations at the non-injured site of 3.2 and 2.8 folds, respectively (*P*<0.05). Twenty-four hours post injury, the expression of TLR-22 was significantly up-regulated by 5 folds at the site of injury relative to control fish (*P*<0.05).

## Discussion

Previous studies have shown that mammalian fibroblasts have immune regulatory capabilities due to their ability to express cytokines and immune receptors on their surface and their reactivity to pathogen associated molecular patterns (PAMPs) [12], [13]. In the present study it is demonstrated, by using the rainbow trout as a model, that these characteristics of the fibroblasts are evolutionary old and that Toll-like receptor and cytokine gene orthologoues to the mammalian counterparts are induced following stimulation in fibroblasts from this fish species. Further, it was shown that rainbow trout fibroblasts are reactive to damage associated molecular patterns (DAMPs). *Seong* and *Matzinger* (2004) proposed the DAMP model and according to this, any molecule that is normally not exposed can be a DAMP if it is revealed during, after or because of injury or damage [39]. Hence, in order to show if ‘foreign’ molecules were more potent inducers than ‘self’ in the fibroblasts, the sonicated fibroblasts were separated into debris containing cell-surface fractions and supernatant containing endogenous molecules like mitochondrial DNA and intracellular proteins [39], [66]. Interestingly, both debris and supernatant induced the expression of IL-1β, whereas only the supernatant stimulated IL-10, but IL-8 was not induced at all. However, debris might also contain ‘foreign’ parts of molecules and structures since the interior side of the cell wall is normally not exposed and could explain why debris stimulated the IL-1β expression. Together, this shows that fibroblasts potentially are able to take part in the inflammation in the body and react to signals released from injured cells. Worthy of note, the results also showed that only LPS induced IL-8 expression in fibroblasts *in vitro*, whereas neither fraction of the injured cells or supernatant could do so. Hence, subsequent neutrophil recruitment via IL-8 chemoattraction most likely relies on stimulation by PAMPs rather than by DAMPs. Alternatively, other cell types in the injured tissue could account for the increase in IL-8 expression observed *in vivo*. However, the constitutive expression of IL-8 was higher in the RTHDF cells than in muscle tissue, indicating that the contribution of IL-8 from fibroblasts is not without significance. In this study, the idea of looking into the immune regulating capabilities of fibroblasts *in vivo* was derived from the results that were obtained from the *in vitro* study using the RTHDF cell-line. From this, we hypothesized that local cells in the musculature of the rainbow trout were able to express cytokine genes following tissue damage and hence were able to react to DAMPs. Previously, it has been shown that skeletal muscle tissue in humans produce IL-6, IL-8 and IL-15, and it could be suggested that local fibroblasts contributes to this [67]. By applying the damage model in the rainbow trout, internal controls in muscle tissue from the injured fish opposite to the damaged side could be sampled. This gave a unique opportunity to show whether the responses were local or systemic. An activation of local cells was initiated at the injury site due to a rapid induction four hours post sampling of IL-1β, IL-8 and TGF-β. This did not occur within the internal control site. It seems realistic that an up-regulation earlier than four hours took place since the level of especially IL-8 was high four hours post injury. The *in vitro* experiment supports this idea, since LPS stimulated RTHDF cells expressed IL-1β and IL-8 significantly higher than control cells already one hour after stimulation. Further, in zebrafish an induction of H_2_O_2_ following tail cutting has been observed in the wound a few minutes after cutting, indicating a very rapid activation of local cells [68]. During the evolution from the early vertebrates (fish) to mammals a number of different TLRs have evolved. Fishes comprise a large heterogeneous group with a high degree of evolutionary distance between species, which is also reflected in the numbers and specific types of TLRs present. In the rainbow trout, five different TLRs named 3, 5, 9 and 22 are known, where TLR-22 is only found in fish [61]. In the evolutionary more ‘advanced’ pufferfish *Fugu rubripes* and the zebrafish *Danio rerio*, a complete set of TLRs orthologous to the ten mammalian TLRs is described [69]. In addition, they also express TLR-21 and 22, where TLR-21 is unique to fish [61]. Both the damage model and the fibroblast *in vitro* experiment showed that the TLR-3 and 9 genes were inducible by DAMPs at the site of injury and by the supernatant from the sonicated fibroblasts, but not by the debris. Hence, this was in accordance with the DAMP model proposed by *Seong* and *Matzinger* since endogenous proteins and molecules released following tissue damage were able to trigger the expression of immune genes [39]. Further, LPS induced TLR-3 and 9 expression in the fibroblasts showing that PAMPs could also stimulate these cells. In contrast, TLR-5 was not affected either in mechanically damaged tissue or in stimulated fibroblasts, indicating that this receptor is triggered by other ligands. TLR-22 was induced by mechanical damage in the rainbow trouts, but not expressed in the non-stimulated RTHDF cells. In the mammals, TLR-3 binds dsRNA, but is also a sensor of tissue necrosis during acute inflammation [70]. Our findings in the rainbow trout fibroblasts suggest that this characteristic is evolutionary old. It is not known whether TLR-3 and 9 are endogenous positioned in cells from rainbow trout, as generally seen in mammalian cells. However, although mostly expressed endosomally, TLR-3 have been shown to be surface expressed in human skin and lung fibroblasts, in certain human fibroblast cell lines and on epithelial cells. Whatever might be the case in fish; our results show that these receptors are inducible in the rainbow trout fibroblasts without the cells being able to phagocytise latex beads. This is in contrast to e.g. macrophages and granulocytes in the rainbow trout, which are phagocytic cell-types [48], [71]. Future studies using nano particles could reveal if the RTHDF cells are able to take up smaller particles [72]. Further, the ability of supernatant to induce TLR-9 expression in the fibroblasts showed that this receptor also functions as a sensor of necrosis. In the closely related species Atlantic salmon (*Salmo salar*), an increase in TLR-9 expression after CpG stimulation have been shown in lymphoid tissue indicating a homologue function of this receptor to the mammalian TLR-9 [73]. Since the salmonids lack TLR-2, it might therefore be suggested that TLR-3 and 9 in these species could have a similar role as TLR-2 in the mammals, where this receptor is also a sensor of necrotic cells, while TLR-9 in the mammals recognises bacterial and viral DNA, unmethylated CpG and DNA without CpG motifs [39], [74]. Together, these results indicate that evolutionary fibroblasts are primitive cells, which have maintained the ability to express genes of immune functions, when stimulated. Their function as being positioned in the tissue, as ‘already there’ before the more potent immune cells are recruited, seems advantageous when damage, injury or infection occurs. However, due to their lack of phagocytosing capability it seems that their immune capacity is not fully developed like traditional immune cells such as macrophages and granulocytes.

## References

[1] Kaiser P, Rothwell L, Avery S, Balu S (2004). Evolution of the interleukins.. Developmental and Comparative Immunology.

[2] Martin P, Leibovich SJ (2005). Inflammatory cells during wound repair: the good, the bad and the ugly.. Trends Cell Biol.

[3] Magor BG, Magor KE (2001). Evolution of effectors and receptors of innate immunity.. Developmental and Comparative Immunology.

[4] Sepulcre MP, Alcaraz-Perez F, Lopez-Munoz A, Roca FJ, Meseguer J (2009). Evolution of Lipopolysaccharide (LPS) Recognition and Signaling: Fish TLR4 Does Not Recognize LPS and Negatively Regulates NF-kB Activation.. J Immunol.

[5] Litman GW, Cannon JP, Dishaw LJ (2005). Reconstructing immune phylogeny: new perspectives.. Nat Rev Immunol.

[6] Diegelmann RF, Evans MC (2004). Wound healing: an overview of acute, fibrotic and delayed healing.. Front Biosci.

[7] Murphy KP, Travers P, Walport M, Janeway C (2008). Janeway's immunobiology..

[8] Dinarello CA (1997). Interleukin-1.. Cytokine Growth Factor Reviews.

[9] Kobayashi Y (2008). The role of chemokines in neutrophil biology.. Front Biosci.

[10] Zhang W, Chen H (2002). [The study on the interleukin-8 (IL-8)].. Sheng Wu Yi Xue Gong Cheng Xue Za Zhi.

[11] Hatakeyama J, Tamai R, Sugiyama A, Akashi S, Sugawara S (2003). Contrasting responses of human gingival and periodontal ligament fibroblasts to bacterial cell-surface components through the CD14/Toll-like receptor system.. Oral Microbiol Immunol.

[12] Hosokawa Y, Hosokawa I, Ozaki K, Nakae H, Matsuo T (2005). Increase of CCL20 expression by human gingival fibroblasts upon stimulation with cytokines and bacterial endotoxin.. Clin Exp Immunol.

[13] Chen B, Tsui S, Smith TJ (2005). IL-1 beta induces IL-6 expression in human orbital fibroblasts: identification of an anatomic-site specific phenotypic attribute relevant to thyroid-associated ophthalmopathy.. J Immunol.

[14] Smith RS, Smith TJ, Blieden TM, Phipps RP (1997). Fibroblasts as sentinel cells. Synthesis of chemokines and regulation of inflammation.. American Journal of Pathology.

[15] Alberts B (1989). Molecular biology of the cell.. Garland Pub.

[16] Ruiz C, Perez E, Garcia-Martinez O, Diaz-Rodriguez L, Arroyo-Morales M (2007). Expression of cytokines IL-4, IL-12, IL-15, IL-18, and IFNgamma and modulation by different growth factors in cultured human osteoblast-like cells.. J Bone Miner Metab.

[17] Yoshiura Y, Kiryu I, Fujiwara A, Suetake H, Suzuki Y (2003). Identification and characterization of Fugu orthologues of mammalian interleukin-12 subunits.. Immunogenetics.

[18] Zou J, Grabowski PS, Cunningham C, Secombes CJ (1999). Molecular cloning of interleukin 1 beta from rainbow trout *Oncorhynchus mykiss* reveals no evidence of an ice cut site.. Cytokine.

[19] Ohtani M, Hayashi N, Hashimoto K, Nakanishi T, Dijkstra JM (2008). Comprehensive clarification of two paralogous interleukin 4/13 loci in teleost fish.. Immunogenetics.

[20] Iliev DB, Castellana B, MacKenzie S, Planas JV, Goetz FW (2007). Cloning and expression analysis of an IL-6 homolog in rainbow trout (Oncorhynchus mykiss).. Molecular Immunology.

[21] Sangrador-Vegas A, Lennington JB, Smith TJ (2002). Molecular cloning of an IL-8-like CXC chemokine and tissue factor in Rainbow trout (Oncorhynchus mykiss) by use of suppression subtractive hybridization.. Cytokine.

[22] Zhang DC, Shao YQ, Huang YQ, Jiang SG (2005). Cloning, characterization and expression analysis of interleukin-10 from the zebrarish (*Danio rerion*).. Journal of Biochemistry and Molecular Biology.

[23] Laing KJ, Wang TH, Zou J, Holland J, Hong SH (2001). Cloning and expression analysis of rainbow trout *Oncorhynchus mykiss* tumour necrosis factor-alpha.. European Journal of Biochemistry.

[24] Zou J, Carrington A, Collet B, Dijkstra JM, Yoshiura Y (2005). Identification and bioactivities of IFN-gamma in rainbow trout *Oncorhynchus mykiss*: The first Th1-type cytokine characterized functionally in fish.. Journal of Immunology.

[25] Peatman E, Liu ZJ (2007). Evolution of CC chemokines in teleost fish: a case study in gene duplication and implications for immune diversity.. Immunogenetics.

[26] Wang TH, Hanington PC, Belosevic M, Secombes CJ (2008). Two macrophage colony-stimulating factor genes exist in fish that differ in gene organization and are differentially expressed.. Journal of Immunology.

[27] Campo GM, Avenoso A, Campo S, Angela D, Ferlazzo AM (2006). TNF-alpha, IFN-gamma, and IL-1beta modulate hyaluronan synthase expression in human skin fibroblasts: synergistic effect by concomital treatment with FeSO4 plus ascorbate.. Mol Cell Biochem.

[28] Kurt-Jones EA, Sandor F, Ortiz Y, Bowen GN, Counter SL (2004). Use of murine embryonic fibroblasts to define Toll-like receptor activation and specificity.. J Endotoxin Res.

[29] Dziarski R, Gupta D (2000). Role of MD-2 in TLR2- and TLR4-mediated recognition of Gram-negative and Gram-positive bacteria and activation of chemokine genes.. J Endotoxin Res.

[30] Takeuchi O, Akira S (2001). Toll-like receptors; their physiological role and signal transduction system.. Int Immunopharmacol.

[31] Medzhitov R, Janeway C (2000). Innate immune recognition: mechanisms and pathways.. Immunol Rev.

[32] Nemoto E, Sugawara S, Tada H, Takada H, Shimauchi H (2000). Cleavage of CD14 on human gingival fibroblasts cocultured with activated neutrophils is mediated by human leukocyte elastase resulting in down-regulation of lipopolysaccharide-induced IL-8 production.. J Immunol.

[33] Skaleric U, Manthey CM, Mergenhagen SE, Gaspirc B, Wahl SM (2000). Superoxide release and superoxide dismutase expression by human gingival fibroblasts.. Eur J Oral Sci.

[34] Abraham LC, Dice JF, Lee K, Kaplan DL (2007). Phagocytosis and remodeling of collagen matrices.. Exp Cell Res.

[35] Arora S, Jain J, Rajwade JM, Paknikar KM (2009). Interactions of silver nanoparticles with primary mouse fibroblasts and liver cells.. Toxicol Appl Pharmacol.

[36] Uehara A, Takada H (2007). Functional TLRs and NODs in Human Gingival Fibroblasts.. J Dent Res.

[37] Sugawara S, Sugiyama A, Nemoto E, Rikiishi H, Takada H (1998). Heterogeneous Expression and Release of CD14 by Human Gingival Fibroblasts: Characterization and CD14-Mediated Interleukin-8 Secretion in Response to Lipopolysaccharide.. Infect Immun.

[38] Kyburz D, Rethage J, Seibl R, Lauener R, Gay RE (2003). Bacterial peptidoglycans but not CpG oligodeoxynucleotides activate synovial fibroblasts by toll-like receptor signaling.. Arthritis Rheum.

[39] Seong SY, Matzinger P (2004). Hydrophobicity: an ancient damage-associated molecular pattern that initiates innate immune responses.. Nat Rev Immunol.

[40] Gonzalez SF, Huising MO, Stakauskas R, Forlenza M, Lidy Verburg-van Kemenade BM (2007). Real-time gene expression analysis in carp (*Cyprinus carpio* L.) skin: inflammatory responses to injury mimicking infection with ectoparasites.. Developmental and Comparative Immunology.

[41] Jault C, Pichon L, Chluba J (2004). Toll-like receptor gene family and TIR-domain adapters in *Danio rerio*.. Molecular Immunology.

[42] Rodriguez MF, Wiens GD, Purcell MK, Palti Y (2005). Characterization of Toll-like receptor 3 gene in rainbow trout (*Oncorhynchus mykiss*).. Immunogenetics.

[43] Skjaeveland I, Iliev DB, Zou J, Jorgensen T, Jorgensen JB (2008). A TLR9 homolog that is up-regulated by IFN-gamma in Atlantic salmon (*Salmo salar*).. Developmental and Comparative Immunology.

[44] Purcell MK, Smith KD, Aderem A, Hood L, Winton JR (2006). Conservation of Toll-like receptor signaling pathways in teleost fish.. Comparative Biochemistry and Physiology D-Genomics & Proteomics.

[45] Hordvik I, Grimholt U, Fosse VM, Lie O, Endresen C (1993). Cloning and Sequence-Analysis of cDNAs Encoding the MHC Class-II Beta-Chain in Atlantic Salmon (*Salmo salar*).. Immunogenetics.

[46] Hansen JD, Landis ED, Phillips RB (2005). Discovery of a unique Ig heavy-chain isotype (IgT) in rainbow trout: Implications for a distinctive B cell developmental pathway in teleost fish.. Proceedings of the National Academy of Sciences of the United States of America.

[47] Warr GW (1995). The Immunoglobulin Genes of Fish.. Developmental and Comparative Immunology.

[48] Secombes CJ, Iwama GaN T (1996). The nonspecific immune system: Cellular defences.. The fish immune system: Organism, pathogen, and environment.

[49] Collet B, Secombes CJ (2001). The rainbow trout (Oncorhynchus mykiss) Mx1 promoter - Structural and functional characterization.. European Journal of Biochemistry.

[50] Trobridge GD, Leong JAC (1995). Characterization of a Rainbow-Trout Mx-Gene.. Journal of Interferon and Cytokine Research.

[51] Collet B, Boudinot P, Benmansour A, Secombes CJ (2004). An Mx1 promoter-reporter system to study interferon pathways in rainbow trout.. Developmental and Comparative Immunology.

[52] Wolf K, Quimby MC (1962). Established Eurythermic Line of Fish Cells in Vitro.. Science.

[53] Ossum CG, Hoffmann EK, Vijayan MM, Holt SE, Bols NC (2004). Characterization of a novel fibroblast-like cell line from rainbow trout and responses to sublethal anoxia.. Journal of Fish Biology.

[54] Zou J, Bird S, Truckle J, Bols N, Horne M (2004). Identification and expression analysis of an IL-18 homologue and its alternatively spliced form in rainbow trout (*Oncorhynchus mykiss*).. European Journal of Biochemistry.

[55] Wang TH, Zou J, Cunningham C, Secombes CJ (2002). Cloning and functional characterisation of the interleukin-1 beta 1 promoter of rainbow trout (*Oncorhynchus mykiss*).. Biochimica Et Biophysica Acta-Gene Structure and Expression.

[56] Bowie A, O'Neill LAJ (2000). The interleukin-1 receptor/Toll-like receptor superfamily: signal generators for pro-inflammatory interleukins and microbial products.. Journal of Leukocyte Biology.

[57] Stylianou E, Saklatvala J (1998). Interleukin-1.. International Journal of Biochemistry & Cell Biology.

[58] Secombes CJ, Bird S, Cunningham C, Zou J (1999). Interleukin-1 in fish.. Fish & Shellfish Immunology.

[59] Commins S, Steinke JW, Borish L (2008). The extended IL-10 superfamily: IL-10, IL-19, IL-20, IL-22, IL-24, IL-26, IL-28, and IL-29.. J Allergy Clin Immunol.

[60] Rebl A, Siegl E, Kollner B, Fischer U, Seyfert HM (2007). Characterization of twin toll-like receptors from rainbow trout (Oncorhynchus mykiss): evolutionary relationship and induced expression by Aeromonas salmonicida salmonicida.. Dev Comp Immunol.

[61] Oshiumi H, Tsujita T, Shida K, Matsumoto M, Ikeo K (2003). Prediction of the prototype of the human Toll-like receptor gene family from the pufferfish, Fugu rubripes, genome.. Immunogenetics.

[62] Ma C, Collodi P (1999). Preparation of primary cell cultures from lamprey.. Methods Cell Sci.

[63] Ganassin RC, Bols NC (1996). Development of long-term rainbow trout spleen cultures that are haemopoietic and produce dendritic cells.. Fish & Shellfish Immunology.

[64] Ingerslev HC, Pettersen EF, Jakobsen RA, Petersen CB, Wergeland HI (2006). Expression profiling and validation of reference gene candidates in immune relevant tissues and cells from Atlantic salmon (Salmo salar L.).. Mol Immunol.

[65] Livak KJ, Schmittgen TD (2001). Analysis of relative gene expression data using real-time quantitative PCR and the 2(T)(-Delta Delta C) method.. Methods.

[66] Alberts B (2008). Molecular biology of the cell..

[67] Nielsen AR, Pedersen BK (2007). The biological roles of exercise-induced cytokines: IL-6, IL-8, and IL-15.. Appl Physiol Nutr Metab.

[68] Niethammer P, Grabher C, Look AT, Mitchison TJ (2009). A tissue-scale gradient of hydrogen peroxide mediates rapid wound detection in zebrafish.. Nature.

[69] Meijer AH, Gabby Krens SF, Medina Rodriguez IA, He S, Bitter W (2004). Expression analysis of the Toll-like receptor and TIR domain adaptor families of zebrafish.. Mol Immunol.

[70] Cavassani KA, Ishii M, Wen H, Schaller MA, Lincoln PM (2008). TLR3 is an endogenous sensor of tissue necrosis during acute inflammatory events.. J Exp Med.

[71] Ganassin RC, Bols NC (1998). Development of a monocyte/macrophage-like cell line, RTS11, from rainbow trout spleen.. Fish & Shellfish Immunology.

[72] Shan X, Liu C, Yuan Y, Xu F, Tao X (2009). In vitro macrophage uptake and in vivo biodistribution of long-circulation nanoparticles with poly(ethylene-glycol)-modified PLA (BAB type) triblock copolymer.. Colloids Surf B Biointerfaces.

[73] Strandskog G, Skjaeveland I, Ellingsen T, Jorgensen JB (2008). Double-stranded RNA- and CpG DNA-induced immune responses in Atlantic salmon: comparison and synergies.. Vaccine.

[74] Kumagai Y, Takeuchi O, Akira S (2008). TLR9 as a key receptor for the recognition of DNA.. Adv Drug Deliv Rev.

